# Knowledge, use and perceptions of artificial intelligence Chatbots among Italian physiotherapists: an online cross-sectional survey

**DOI:** 10.3389/fdgth.2025.1671521

**Published:** 2025-09-08

**Authors:** Manuela Deodato, Raffaele Sabot, Alessandra Galmonte, Alvisa Palese, Greta Castellini, Andrea Turolla, Paolo Pillastrini, Chad Cook, Lia Rodeghiero, Silvia Gianola, Giacomo Rossettini

**Affiliations:** ^1^ Department of Medicine, Surgery and Health Sciences, University of Trieste, Trieste, Italy; ^2^ Department of Medical Sciences, University of Udine, Udine, Italy; ^3^ Unit of Clinical Epidemiology, IRCCS Istituto Ortopedico Galeazzi, Milan, Italy; ^4^ Department of Biomedical and Neuromotor Sciences—DIBINEM, Alma Mater Studiorum Università di Bologna, Bologna, Italy; ^5^ Unit of Occupational Medicine, IRCCS Azienda Ospedaliero-Universitaria di Bologna, Bologna, Italy; ^6^ Department of Orthopaedics, Duke University, Durham, NC, United States; ^7^ Duke Clinical Research Institute, Duke University, Durham, NC, United States; ^8^ Department of Population Health Sciences, Duke University, Durham, NC, United States; ^9^ Department of Rehabilitation, Hospital of Merano (SABES-ASDAA), Teaching Hospital of Paracelsus Medical University (PMU), Merano-Meran, Italy; ^10^ School of Physiotherapy, University of Verona, Verona, Italy; ^11^ Department of Physiotherapy, Faculty of Sport Sciences, Universidad Europea de Madrid, Villaviciosa de Odón, Spain

**Keywords:** chatGPT, large language model (LLM), chatbot, rehabilitation, pain, physiotherapy (MeSH), artificial intelligence—AI, survey

## Abstract

**Introduction:**

Artificial Intelligence (AI) Chatbots are increasingly being integrated into healthcare, but little is known about their role in physiotherapy. This study investigated the knowledge and use, perceived benefits, limits, and barriers of AI Chatbots in the Italian physiotherapy community.

**Methods:**

A cross-sectional survey was conducted between March and July 2024. Italian physiotherapists, members of the Associazione Italiana di Fisioterapia (AIFI), were invited through mailing lists and social media. Inclusion criteria: AIFI membership, current employment as a physiotherapist, Italian language proficiency, and willingness to participate. A total of 415 out of 2,773 physiotherapists responded (15% response rate); 50.6% were women, and 50.4% had more than 10 years of experience. The survey comprised four sections: (a) respondent characteristics; (b) knowledge and use of AI Chatbots; (c) perception of benefits; and (d) perception of limits and barriers. Descriptive statistics and multivariable logistic regression analyses were performed.

**Results:**

Overall, 93.3% of physiotherapists had heard of AI Chatbots, but 66.9% had never used them in clinical practice. Among those who had, 11.3% reported a “positive” and 14.5% a “very positive” experience. Despite limited use, 78% expressed a positive attitude towards future adoption, and 50% considered AI Chatbots potentially helpful in clinical practice. Reported risks included patient self-diagnosis (84.4%), spread of false information (72.1%), and reduced human interaction (64%). Having more than 21 years of experience was significantly associated with a higher frequency of AI Chatbot use (OR: 5.93, *p* = 0.013). Age was also a significant predictor of use frequency (OR: 1.05, *p* = 0.013), with older physiotherapists reporting more frequent AI use.

**Conclusion:**

Italian physiotherapists acknowledged both opportunities and risks in implementing AI Chatbots. Although current adoption is limited, the overall positive attitude suggests a likely increase in future use. Targeted strategies, including guidelines and educational initiatives, are needed to ensure safe and effective integration into clinical practice.

## Introduction

Artificial intelligence (AI) is a branch of advanced technology that performs tasks that usually require human intelligence (1). In this emerging field, AI Chatbots (e.g., ChatGPT, Google Gemini, Microsoft Copilot, Bing Chat, YouChat, and Google Bard) have been established as innovative language models capable of generating coherent, human-like responses, using text or speech, through algorithms designed to comprehend natural human language (2, 3). Recently, the interest around AI Chatbots has extended to the healthcare field, including physiotherapy (4, 5).

Several studies have examined the potential support that AI Chatbots could provide in various areas of physiotherapy clinical practice (4–6). AI Chatbots could streamline clinical workflow by assisting physiotherapists in extracting information from patients' clinical notes (7) and designing exercise programs (8, 9). Additionally, AI Chatbots could aid in clinical reasoning and decision-making processes by triaging patients' symptoms and suggesting appropriate physiotherapy tests and rehabilitation goals (10, 11). Moreover, AI Chatbots could enhance patient education by offering accessible information regarding conditions and treatment options to improve adherence and motivation to physiotherapy (12, 13).

Although AI Chatbots show promise, their adoption in physiotherapy clinical practice is wrought with limitations and ethical issues (4–6). One concern is the potential of privacy violations and unauthorized access to sensitive patient data if appropriate protections are not adopted (5). Additionally, there are risks of AI providing outdated or imprecise information, thus resulting in treatment recommendations that do not match evidence-based practice guidelines (5). The use of AI Chatbots could automate and dehumanize interactions, threatening the physiotherapist's professional encounter with patient, which requires relational, cognitive and physical skills that AI algorithms cannot replicate (5, 14, 15). Consequently, development of guidelines that outline the pros and cons of AI Chatbots is recommended (5).

Several surveys (16–26) and one qualitative study (27) have analyzed the use, perception, knowledge, and attitudes of healthcare professionals regarding AI Chatbots, mainly focusing on ChatGPT. Prior work has found statistically significant associations between the age of the responders and their attitudes towards using ChatGPT) (17, 22, 28), as well as between familiarity with ChatGPT and the interest in utilizing these platforms (17, 20, 28). Other associations were observed between the belief that ChatGPT could improve patient outcomes and enhance the future of healthcare (20), between the inclination to use ChatGPT in the future and the belief that it could improve medical research, as well as perceived potential benefits and awareness of their potential concerns (20). This preliminary evidence highlights the need to continue investigating AI Chatbots across various healthcare professions and international contexts to identify potential cross-country and interprofessional differences.

To date, studies have mainly focused on certain healthcare professionals (e.g., nurses, technicians, physicians) from regions such as the Middle East (e.g., Jordan, Saudi Arabia, Egypt), Asia (e.g., China and Thailand), the Americas (e.g., USA and Latin America), and Europe (e.g., France and UK) (16–25, 28). Our study offers the opportunity to extend this line of research to underexplored contexts, such as Italy, and to include rehabilitation professionals like physiotherapists. Despite previous studies on the perception, knowledge, and attitudes of physiotherapists concerning AI machine learning (29–31), no surveys have been conducted on AI Chatbots conducted among physiotherapists. Our study aims to investigate the knowledge and use, perception, limits and barriers regarding AI Chatbots among Italian physiotherapists, and will investigate the odds of use across selected sample characteristics. This approach expands the current evidence by introducing perspectives from a country and a profession, both deeply grounded in physical contact as a primary mode of interaction.

## Materials and methods

### Study design and ethics

A quantitative cross-sectional survey was conducted on Italian Physiotherapists in accordance with the Checklist for Reporting Results of Internet E-Surveys (CHERRIES) guidelines (32), STrengthening the Reporting of OBservational Studies in Epidemiology (STROBE) guidelines (33) and the Consensus-Based Checklist for Reporting of Survey Studies (CROSS) (34). The study was approved by the institutional review board of University of Trieste (number 45374; approved on 26/02/2024), and was prospectively registered on OSF database (https://osf.io/zskgy/).

### Participants and setting

The target population was a sample of Italian physiotherapists members of Associazione Italiana di Fisioterapia (AIFI, Rome Italy) (35). AIFI, established in 1959, is a national technical-scientific association dedicated to physiotherapy science, with the mission of promoting the scientific, ethical, and human growth of physiotherapists to benefit individuals and the community (35). At the time of the survey, AIFI had 2,773 active registered physiotherapists. We included participants if they met the following criteria: (a) AIFI membership, (b) current employment as a physiotherapist, (c) ability to understand the Italian language, and (d) willingness to participate. Students were excluded from the study.

### Sample size

The SurveyMonkey sample size calculator (36) was used to estimate the number of responses required for the study. Based on a population size of 2,773 AIFI physiotherapists members at the time of the survey, a 5% margin of error (suggesting how closely the survey results reflect the views of the overall population), and a 95% confidence level (suggesting how confident we can be that the population would select an answer within a certain range), the calculated sample size for completed responses was 338.

### Questionnaire survey development and pre-testing

The open survey on AI Chatbots was developed using the existing studies on healthcare professionals available at the time of the project conception (16–25, 27, 28). A multi-professional team of six clinicians and researchers from various healthcare fields (e.g., physiotherapy, nursing, and epidemiology) developed and critically evaluated the survey for face and content validity (37). Two AIFI members were also involved in this process. Upon completion, only linguistic adjustments were made, resulting in a total of 22 questions distributed over ten web pages.

Next, the survey was piloted with 15 AIFI physiotherapists from different regions of Italy (North, *n* = 5; Center, *n* = 5; South of Italy, *n* = 5, male/female = 7/8, mean age ± standard deviation = 36 ± 9), all of whom were AIFI members. The pilot data was not included in the final data set of the study. According to the International Handbook of Survey Methodology (37), a telephone debriefing session was conducted with the pilot sample to assess potential issues with the survey. The pilot sample reported that the questions were clear and easy to understand; thus, no further changes were needed.

### Questionnaire administration

The final version of the questionnaire consisted of four sections. In section 1 (“Respondent characteristics”), we collected data regarding the socio-demographic, professional, and educational variables of the sample with 11 questions (one open-ended, single-answer question and ten closed, multiple-choice questions). Section 2 (“Knowledge and use”) comprised eight closed, multiple-choice questions exploring knowledge of AI Chatbots, their use and experience in clinical practice, and attitudes towards future use. Section 3 (“Perceptions”) was composed of two closed, multiple-choice questions. The questions focused on the perceived benefits of AI Chatbots in clinical practice and their potential applications, using a Likert scale, ranging from 0 (“Not at all useful”) to 4 (“Extremely useful”) and from 0 (“Strongly disagree”) to 4 (“Strongly agree”). Finally, in Section 4 (“Limits and barriers”), we considered the limits and barriers of AI Chatbots in clinical practice with one closed, multiple-choice question, using a Likert scale ranging from 0 (“Strongly disagree”) to 4 (“Strongly agree”). Both the English and Italian versions of the questionnaire are provided in Supplementary 1 and 2, respectively.

### Data collection procedure

Survey Monkey (1) (Survey Monkey Company, Palo Alto, CA, USA 2024) (38), an online survey tool, was used for data collection. The open survey was administered between March 27th and July 16th, 2024. The mailing list and social media channels of AIFI were used to contact participants. The communication included the survey link (https://it.surveymonkey.com/r/AI_Fisioterapisti_Italiani), the study's aim, details on data handling procedures (anonymity), an informed consent statement, and an invitation to complete the survey. Specifically, the email informed participants that by clicking on the survey link, they consented to participate in the study (2) (37). Three reminders on email and social media were sent, with the first one a month after the first contact, to encourage participation (39).

To optimize the response rate, the survey was designed to take 10–15 min to complete (40). A SurveyMonkey feature was activated to prevent multiple responses from the same IP address. Participation was voluntary, no incentives were offered, and participants had the option to change their responses or skip specific questions or the entire questionnaire (37). The identity of all participants was protected with a de-identification of sensitive data. Moreover, data were only accessible to the principal investigator, who downloaded and stored it on an encrypted computer (37).

### Variables

The questionnaire adopted multiple-choice and Likert-scale questions (41), that assessed the increased or decreased odds of use and perception. Demographic characteristics (e.g., sex, age, working setting, time dedicated to work, years of experience, higher education) were grouped into categories, for example, working hours (16–30; 31–45; 46–60; >60).

We dichotomized three questions: question 15 (“If yes, how was your experience with AI Chatbots in clinical practice?”), question 17 (“If yes, how often do you use AI Chatbots for clinical purposes on average?”), and question 18 (“How likely are you to use AI Chatbots in the future for clinical purposes?”). For question 15, “very negative,” “negative,” and “neutral” were grouped together vs. “positive” and “very positive”; for questions 17 and 18, “never,” “rarely,” and “sometimes” were combined vs. “often” and “always”. An automated response rate count was obtained for each of the four sections to identify sample size and any early terminations (i.e., responders who did not complete all four sections). Questionnaires with early terminations were excluded from the analyses (respondents who failed to complete later sections, such as Section 3).

### Statistical analyses

Data were exported from SurveyMonkey and analyzed using STATA version 17 (42). Descriptive statistics were adopted to summarize the participant characteristics and questionnaire responses. Continuous variables were summarized using the median and interquartile range (IQR), while categorical variables were summarized using absolute frequencies and the corresponding percentages. To assess the relationship between participants' demographic characteristics (e.g., sex, age, working setting, time dedicated to work, years of experience, higher education) and their perceptions of AI Chatbots, a multivariable logistic regression analysis was performed. Specifically, Questions 15, 17, and 18 (dichotomized and described previously) were used as dependent variables within the models. All models were adjusted for potential confounders, including demographic variables, and statistical significance was set at *p* < 0.05 for all analyses.

## Results

### Response rate and respondent characteristics

Four hundred and fifteen out of 2,773 physiotherapists (response rate of 15%) participated in the survey. The participants had a mean age of 37 years (SD 9.8). Half of the participants were women (50.6%), and 50.4% had over 10 years of experience in physiotherapy. Most lived in the North of Italy (67.2%), worked in the private sector (68.4%), and were employed as physiotherapists (53%). Most respondents worked in outpatient settings (67%) and primarily treated adult patients (76.9%) in the musculoskeletal area (72.8%), with weekly work hours ranging from 31 to 45 h (65.8%). The majority held a postgraduate diploma/certification (48.6%) (Table 1).

**Table 1:** General characteristics of the sample.

Variables	Characteristics	*N*	%
Age (Mean and sd)		37	9.8
Sex			
	Women	210	50.6
	Men	203	48.9
	Non-binary	0	0
	I don't want to answer	2	0.5
Years of experience as PT			
	<5	83	20
	6–10	123	29.6
	11–20	80	19.3
	>21	129	31.1
Italy			
	North	279	67.2
	Centre	69	16.6
	South	67	16.2
Working Sector			
	Private	284	68.4
	Public	131	31.6
Professional Status			
	Employed physiotherapists	220	53
	Freelancer	195	47
Work Setting			
	Outpatients	278	67
	Hospitals	101	24.3
	Community	36	8.7
Type of Treated Patients			
	Adults	319	76.9
	Elderly	78	18.8
	Paediatrics	18	4.3
Primary Field of Practice			
	Musculoskeletal	302	72.8
	Neurologic	79	19
	Cardio-respiratory	18	4.3
	Uro-gynaecological	10	2.4
	Oncologic-lymph	6	1.5
Hours worked in a week			
	1–15	10	2.4
	16–30	55	13.3
	31–45	273	65.8
	46–60	69	16.6
	>60	8	1.9
Highest Level of Education			
	Postgraduate diploma/certification	202	48.6
	Bachelor’ degree	118	28.4
	Master’s degree	81	19.5
	PhD	14	3.5

*N*, numbers; %, percentage; SD, standard deviations; <, minor; >, major; PhD, doctor of philosophy; PT, physiotherapist.

### Knowledge and use

Overall, 93.3% of the sample had heard of AI Chatbots (e.g., ChatGPT, Microsoft Copilot, and Google Gemini) through various sources, including family, friends or colleagues (36.1%), social media (33.7%), traditional media (13.3%), scientific articles (6.3%), and university lectures (3.9%). The majority had never used an AI Chatbot in clinical practice (66.9%). Among those who had, 11.3% reported a “positive” and 14.5% a “very positive” experience. The main reported functions included searching for strategies to treat patients (12.8%), obtaining suggestions for education interventions (11.1%), conducting medical history (8.7%), and developing strategies for patient empowerment (7%), among other uses. Only a small percentage of participants reported using AI Chatbots “often” for clinical purposes (8.4%), while half expressed interest in using them “sometimes” in the future (52.8%). Additionally, most participants considered AI Chatbots to be neither easy nor difficult to use (45.1%) (Table 2).

**Table 2:** Knowledge and use of AI Chatbots.

Items	Total	*N*	%
“Have you ever heard about AI Chatbots (i.e., ChatGPT, Google Bard, Bing)?”	Yes	387	93.3
	No	28	6.7
“If yes, in what context did you become aware of the existence of AI Chatbots?”	Friends/family/colleagues	150	36.1
	Social media	140	33.7
	Traditional media (TV/Newspapers)	55	13.3
	Never heard about it	28	6.7
	Scientific articles	26	6.3
	University lectures	16	3.9
	Other	0	0
“Have you ever used AI Chatbots in clinical practice?”	Yes	137	33.1
	No	278	66.9
“If yes, how was your experience with AI Chatbots in clinical practice?”	Never use it	278	67
	Negative	3	0.7
	Neutral	27	6.5
	Positive	47	11.3
	Very Positive	60	14.5
“If yes, for what purpose have you mainly used AI Chatbots in clinical practice?”^a^	Medical history	36	8.7
	Clinical reasoning	14	3.4
	Functional diagnosis	14	3.4
	Management of medical records	11	2.7
	Identify strategies for treating the patient’s problems	53	12.8
	Identify empowerment strategies to encourage patient self-care	29	7.0
	Provide support in the interpretation of clinical data, test results or assessments	27	6.5
	Track down answers to use in educational interventions with the patient	46	11.1
	Never used AI chatbots for clinical purposes	285	68.7
	Other	0	0
“If yes, how often do you use AI Chatbots for clinical purposes on average?”	Never	286	68.9
	Rarely (very low frequency)	49	11.8
	Sometimes	44	10.6
	Often (moderate frequency)	35	8.4
	Always	1	0.2
“How likely are you to use AI Chatbots in the future for clinical purposes?”	Never	20	4.8
	Rarely (very low frequency)	77	18.6
	Sometimes	219	52.8
	Often (moderate frequency)	95	22.9
	Always	4	1
“How easy or difficult do you consider using AI Chatbots?”	Very difficult	7	1.7
	Difficult	44	10.6
	Neither easy, nor difficult	187	45.1
	Easy	149	35.9
	Very Easy	28	6.7

^a^ Multiple answers were allowed and percentage are reported related to the total numbers of participants (*n* = 415)

*N*, number; %, percentage; AI, artificial intelligence.

### Perceptions, limits and barriers

Regarding the perceived benefits of AI Chatbots in clinical practice and their potential applications, the highest number of positive responses were relative to scheduling appointments (median: 3 out of 4), managing patients' medical records (median: 3 out of 4), creating online content for patients' home exercise (median: 3 out of 4), and generating social media posts (median: 3 out of 4), (Table 3). In addition, participants expressed moderate agreement with the usefulness of AI Chatbots: many considered them helpful in providing access to accurate and up-to-date information and supportive in clinical practice (median = 2 out of 4). However, fewer participants agreed that AI Chatbots are more useful than established resources such as search engines or databases (e.g., PubMed) (median = 2 out of 4), (Table 4). Concerning the limits and barriers of AI Chatbots, physiotherapists reported the following significant risks: patient self-diagnosis (median: 3 out of 4), dissemination of misinformation (median: 3 out of 4), reduction of human interactions (median: 3 out of 4), validity of the content (median: 3 out of 4) (Table 5).

**Table 3:** Perception: do you think artificial intelligence Chatbots are useful in the clinical practice?

Items	Likert Score Median (IQR)	4) Extremely useful, *n* (%)	3) Very useful, *n* (%)	2) Somewhat useful, *n* (%)	1) Not very useful, *n* (%)	0) Not at all useful, *n* (%)
Support during history taking	2 (1–2)	8 (1.9)	89 (21.4)	148 (35.7)	104 (25.1)	66 (15.9)
Support in clinical reasoning and functional diagnosis	2 (1–2)	5 (1.2)	86 (20.7)	174 (41.9)	84 (20.2)	66 (15.9)
Treatment planning for patients	2 (1–2)	5 (1.2)	87 (21)	138 (33.2)	114 (27.5)	71 (17.1)
Appointment schedule planning	3 (2–3)	83 (20)	153 (36.9)	97 (23.4)	59 (14.2)	23 (5.5)
Medical record management	3 (2–4)	108 (26)	116 (27.9)	114 (27.5)	59 (14.2)	18 (4.3)
Analyzing patient data and providing personalized treatment recommendations based on their medical history	2 (1–2)	20 (4.8%)	66 (15.9%)	212 (51.1%)	96 (23.1%)	21 (5%)
Creating online content to give patients to perform exercises at home	3 (2–4)	114 (27.5)	144 (34.7)	115 (27.7)	36 (8.7)	6 (1.4)
Assist in content creation by generating ideas for social media posts and other marketing materials	3 (2–4)	113 (27.2)	136 (32.8)	124 (29.9)	33 (7.9)	9 (2.1)
Processing invoices or bill payments	2 (1–3)	35 (8.4)	152 (36.6)	117 (28.2)	88 (21.2)	23 (5.5)
Identifying empowerment strategies to foster patient self-care	2 (2–3)	20 (4.8)	109 (26.3)	192 (46.3)	79 (19)	15 (3.6)
Tracking answers for use in educational interventions with the patient	1 (1–3)	19 (4.6)	115 (27.7)	0 (0)	262 (63.1)	19 (4.5)

*N*, number; %, percentage; IQR, interquartile range.

**Table 4:** Perception: what is your level of agreement with the following statements about artificial intelligence Chatbots?

Items	Likert Score Median (IQR)	4) Strongly agree, *n* (%)	3) Agree, *n* (%)	2) Neither agree nor disagree, *n* (%)	1) Disagree, *n* (%)	0) Strongly disagree, *n* (%)
The use of AI Chatbots is helpful in providing access to accurate and up-to-date information	2 (1–3)	14 (3.4)	128 (30.8)	147 (35.4)	114 (27.5)	12 (2.9)
AI Chatbots can help me in my clinical practice	2 (2–3)	41 (9.9)	165 (39.7)	123 (29.6)	76 (18.3)	10 (2.4)
AI Chatbots are more useful than search engines (e.g., Google) or databases (e.g., PubMed)	2 (1–2)	12 (2.8)	64 (15.4)	150 (36.1)	128 (30.8)	61 (14.6)

*N*, number; %, percentage; IQR, interquartile range; AI, artificial intelligence.

**Table 5:** Limits and barriers: how much do you agree on the limits of artificial intelligence Chatbots in clinical practice?

Items	Likert score median (IQR)	4) Strongly agree, *n* (%)	3) Agree, *n* (%)	2) Neither agree nor disagree, *n* (%)	1) Disagree, *n* (%)	0) Strongly disagree, *n* (%)
Validity of inaccurate, out-of-date content	3 (2–3)	76 (18.3)	168 (40.4)	148 (35.6)	23 (5.5)	0 (0)
Privacy and Ethical Issues	2 (2–3)	41 (9.8)	125 (30.1)	205 (49.3)	38 (9.1)	6 (1.4)
Risk of dissemination of misinformation	3 (2–4)	127 (30.6)	172 (41.5)	106 (25.5)	10 (2.4)	0 (0)
Reduction in human interactions	3 (2–4)	132 (31.8)	134 (32.2)	69 (16.6)	71 (17.1)	9 (2.1)
Risk of self-diagnosis by patients	3 (2–4)	227 (54.6)	124 (29.8)	48 (11.5)	16 (3.8)	0 (0)
Reduction in quality of patient care	2 (1–3)	75 (18)	98 (23.6)	127 (30.6)	62 (14.9)	53 (12.7)
Harmful or incorrect clinical decisions	2 (2–3)	33 (7.9)	112 (26.9)	203 (48.9)	63 (15.1)	4 (0.9)
Lack of personalized treatment	2 (1–3)	72 (17.3)	116 (27.9)	99 (23.8)	76 (18.3)	52 (12.5)

N, number; %, percentage; IQR, interquartile range.

### Increased or decreased use and perceptions

The analysis of the association between demographic characteristics and participants' perceptions and use of AI Chatbots revealed several findings (Table 6). There is no significant association between age and the evaluation of experience with AI Chatbots (OR: 0.99, 95% CI: 0.95–1.04, *p* = .93) or the likelihood of future use (OR: 1.00, 95%CI: 0.94–1.06, *p* = .86). A significant association is observed with current usage frequency (OR: 1.05, 95%CI: 1.01–1.10, *p* = .01), suggesting that older individuals might use AI Chatbots more frequently for clinical purposes. In terms of years of experience, participants with over 21 years of experience were significantly more likely to use AI Chatbots for clinical purposes (OR: 5.93, 95%CI: 1.45–24.24, *p* = 0.013), compared to those with fewer than 5 years of experience. However, no significant associations were found between sex, working sector, working hours, or education level and the evaluation of experience, frequency of use, or future likelihood of using AI Chatbots.

**Table 6:** Association between characteristics and perceptions and use.

Variables	Question 15			Question 17			Question 18		
	“If yes, how was your experience with AI Chatbots in clinical practice?”			“If yes, how often do you use AI Chatbots for clinical purposes on average?”			“How likely are you to use AI Chatbots in the future for clinical purposes?”		
	OR	CI	*p* value	OR	CI	*p* value	OR	CI	*P* value
Age (years)	0.99	0.95–1.04	0.93	**1** **.** **05**	**1** **.** **01–1** **.** **10**	**0** **.** **013**	1.00	0.94–1.06	0.86
Sex									
male (ref: female)	1.71	0.75–3.89	0.19	1.41	0.70–2.84	0.32	2.14	0.74–6.15	0.15
Years of experience									
6–10 (ref > 5)	1.53	0.53–4.38	0.42	1.34	0.56–3.20	0.50	0.92	0.24–3.55	0.90
11–20 (ref > 5)	1.16	0.39–3.48	0.78	2.27	0.86–5.96	0.09	1	0.22–4.36	1.00
> 21 (ref: >5)	0.9	0.25–3.29	0.87	**5** **.** **93**	**1** **.** **45–24** **.** **24**	**0** **.** **013**	1.03	0.17–6.25	0.97
Public (ref: private sector)	1.00	0.38–2.61	0.99	0.98	0.43–2.21	0.96	0.60	0.19–1.88	0.38
Working hours									
16–30 (ref: 1–15)	1	0.14–7.09	1	0.40	0.05–2.77	0.35	1.6	0.11–21.58	0.72
31–45 (ref: 1–15)	1.68	0.28–9.91	0.56	0.65	0.11–3.76	0.63	1.46	0.15–13.80	0.74
46–60 (ref: 1–15)	4	0.50–31.98	0.19	1.42	0.21–9.58	0.71	2.5	0.18–33.17	0.48
>60 (ref: 1–15)	1	0.05–18.91	1	0.25	0.01–4.72	0.35	0.4	0.01–10.01	0.57
Education									
MSc (ref:BSc)	0.98	0.33–2.88	0.97	1.24	0.45–3.42	0.67	1.84	0.44–7.62	0.39
Postgraduate diploma/certification (ref:BSc)	2.2	0.76–6.35	0.14	0.80	0.32–1.98	0.63	3.31	0.81–13.43	0.09
PhD (ref:BSc)	0.80	0.12–5.26	0.81	0.32	0.05–2.07	0.23	0.21	0.03–1.41	0.11

For question 15, “very negative,” “negative,” and “neutral” were grouped together vs. “positive” and “very positive”; for questions 17 and 18, “never,” “rarely,” and “sometimes” were combined vs. “often” and “always”—

In bold the statistically significance.

OR, odds ratio; Sd, standard deviation; >, major; AI, artificial intelligence; BSc, bachelor’ degree; MSc, master’s degree; PhD, doctor of philosophy.

## Discussion

### Main findings

This study found that age and years of professional experience were positive predictors of the current frequency of AI Chatbot use among Italian physiotherapists. Older clinicians and those with >21 years of experience reported higher odds of current clinical use. These findings contrast with some previous studies, which reported higher levels of engagement and use among younger professionals (17, 20, 22, 28), but are consistent with Hu et al. (21), who linked more years of experience and higher education to positive attitudes toward AI Chatbots (21). It is plausible that senior physiotherapists perceive AI Chatbots as valuable resources to enhance their knowledge, remain updated, and reduce administrative workload (5). This highlights the importance of further exploring the underlying factors that facilitate or hinder adoption among younger professionals and those with less clinical experience (5).

Moreover, the study showed that physiotherapists, regardless of other demographic characteristics (e.g., sex, work setting, or clinical field), expressed a generally positive attitude toward the future use of AI Chatbots, recognizing several potential benefits. At the same time, participants highlighted risks associated with their use, particularly the automation and possible dehumanization of physiotherapy, a profession that requires relational, cognitive, and manual skills that algorithms cannot replicate. These findings suggest that physiotherapists are currently in an exploratory phase in their engagement with AI Chatbots, weighing their potential advantages and limitations, similarly to other healthcare professionals (16–25, 27, 28).

### Comparison with evidence

Regarding knowledge and use, although nearly all Italian physiotherapists had heard of AI Chatbots, a majority had never used them, and only a small minority reported frequent clinical use (8.4%), a pattern consistent with findings among other healthcare professionals (16–28). Most respondents first learned about Chatbots through informal channels (colleagues, family, social media), whereas relatively few cited scientific articles or university teaching, suggesting a gap in structured education on AI in physiotherapy that may shape expectations and perceptions (5).

Despite limited current use, participants expressed a positive attitude toward future adoption and identified practical benefits, including reduced administrative workload, support for patient-education content and social media, and assistance with records and appointment management (16, 17, 24, 25). In line with perspectives reported by radiologists (18), nurses (26), physicians (27) and psychiatrists (25), physiotherapists viewed AI Chatbots as potentially supportive tools for clinical reasoning. However, perceived usefulness was only moderate, indicating that AI Chatbots are regarded as supplementary aids rather than primary decision-making tools (5). By contrast, pharmacists (23), physicians (16, 17) and plastic surgeons (24) reported greater scepticism about personalized treatment and recommendations based on patient data.

Participants also recognize limits and barriers to using AI Chatbots in clinical practice, including risk of patient self-diagnosis, reduced human connection, and misinformation. These concerns mirror those of other healthcare professionals and reflect apprehension that delegating complex tasks to AI Chatbots could erode essential human expertise (16). In physiotherapy, the issue is particularly critical given the reliance on both hard skills (e.g., hands-on techniques and differential diagnosis triage) and soft skills (e.g., empathy and communication) (43), which algorithms cannot replicate (15). In addition to these concerns, another barrier relates to usability: half of physiotherapists rated AI Chatbots as “neither easy nor difficult to use,” a neutral stance likely reflecting limited familiarity or insufficient training rather than inherent technological barriers, in line with available evidence from other healthcare professions where similar patterns have been observed (19, 20, 22).

### Implications for education and research

Given the inevitable integration of AI Chatbots into our daily and professional lives, the physiotherapy community should be prepared to navigate the opportunities and risks associated with their use in practice. This study offers implications for clinical practice and research to support this transition effectively. In clinical practice, two actions should be prioritized. First, it is essential to develop guidelines for the use of AI Chatbots in physiotherapy (5). Recent European guidelines were designed to mitigate the risks and enhance the benefits of AI application (44, 45). In light of the positive attitude expressed by Italian physiotherapists toward the future use of AI Chatbots, these guidelines could be adapted specifically for the field of physiotherapy. This would allow integration of AI Chatbots across different stages of rehabilitation (e.g., history taking, physical examination, treatment administration, and prognosis), while ensuring adherence to ethical and professional standards, and safeguarding patient data privacy and security. Second, training courses on AI Chatbots are needed (5), especially considering that most physiotherapists in our sample have never used them. Educational initiatives would help physiotherapists understand the potential, limitations, and risks of AI Chatbots, promoting responsible adoption. In this regard, Italian professional associations and regulatory bodies, such as AIFI (35) and the National Professional Register of Physiotherapy (FNOFI—Federazione Nazionale Ordini Fisioterapisti, Rome, Italy) (46) could work together to support the implementation process.

Future research should explore the knowledge and use, perceptions, limits and barriers of AI Chatbots among physiotherapists specializing in various branches of physiotherapy, including musculoskeletal, neurological, pediatric, geriatric, lymphatic, and pelvic floor rehabilitation. Furthermore, cross-national and cross-continental surveys should be conducted to assess whether socio-economic, cultural, and educational differences influence physiotherapists' views on AI Chatbots. Finally, the use of qualitative studies (e.g., interviews and focus groups) would be valuable in understanding physiotherapists' lived experiences with AI Chatbots (5, 47).

### Strengths and limitations

As a strength, this is the first study that sheds light on the emerging perceived role of AI Chatbots in the physiotherapists' clinical practice. The study was developed and conducted in adherence to international reporting guidelines (STROBE, CHERRIES and CROSS) (32–34), ensuring methodological rigor. Moreover, the survey, compared to other study designs (e.g., focus groups), by adopting various types of questions (e.g., single and multiple choice), increases the likelihood of adequately capturing the multifaceted nature of the phenomenon under study (48).

Some limitations should be acknowledged. Firstly, although the number of respondents exceeded the required *a priori*-determined sample size, the response rate was lower compared to other studies (16, 17, 21, 22, 24, 26, 28), which could limit the generalizability of our findings to the full Italian physiotherapist population. Secondly, since the survey relied on self-report, recall bias and social desirability bias may have affected the validity of the results (48). Thirdly, merging neutral and disagreement responses into a single category, on one hand, may have reduced data granularity and limited interpretive nuance; on the other hand, this aimed to emphasize clear agreement, reduce ambiguity, and enhance contrast (49). However, this pragmatic option may have influenced how participants' attitudes were distributed and interpreted (49). Finally, because recruitment occurred via mailing lists and social media, we lack information on non-respondents, limiting the assessment of potential non-response bias.

## Conclusion

The findings reveal a positive attitude among Italian physiotherapists towards the integration of AI Chatbots into clinical practice, despite their limited current adoption and declared concerns about risks such as self-treatment, misinformation, and reduced human interaction. Growing interest among clinicians suggests an increase in the adoption and implementation of AI Chatbots in the near future. To ensure a safe and effective transition, targeted strategies should focus on mitigating perceived risks, enhancing awareness of benefits, and providing tailored education and support, particularly for less experienced physiotherapists. Furthermore, as AI Chatbots and algorithms advance, it is essential to develop ethical guidelines, continuously monitor security standards, and ensure the accuracy of the generated information. Aligning these efforts with insights from our survey on knowledge, use, perceived benefits, limits, and barriers will be pivotal in guiding the responsible adoption of AI technologies in physiotherapy.

## Data Availability

The datasets presented in this study can be found in online repositories. The names of the repository/repositories and accession number(s) can be found below: The data that support the findings of this study are available from OSF repository at https://osf.io/zskgy/.
