# Supplementary material for: Knowledge, use and perceptions of artificial intelligence Chatbots among Italian physiotherapists: an online cross-sectional survey
Source: Front Digit Health. 2025 Sep 8;7:1671521. doi: 10.3389/fdgth.2025.1671521 (PMC12450932; doi:10.3389/fdgth.2025.1671521)
Supplement: Supplementary file 1 [file Datasheet1.pdf]

INTRODUCTION

Dear Colleague,

We invite you to participate in a research project aimed at Italian physiotherapists.

The purpose of this study is to conduct a cross-sectional survey using an online questionnaire to investigate the knowledge, barriers, and limitations in the use of artificial intelligence (AI) chatbots. The survey will be distributed to Italian physiotherapists who are officially registered with the FNOFI Professional Order and members of the Italian Physiotherapy Association (AIFI). The study was presented to AIFI president Dr. Simone Cecchetto to distribute to the members' mailing list.
The goal is to investigate the knowledge, applications, potential, limitations, and ethical issues regarding the use of AI chatbots (e.g., ChatGPT, Microsoft Copilot, Google Gemini) in physiotherapy practice. You will be asked to complete a questionnaire that will take a maximum of 10 minutes. Participation in the study is voluntary and poses no specific risks. All collected data will be treated and stored strictly anonymously, in accordance with Legislative Decree 196/2003 and its subsequent amendments regarding personal data protection.
The study will be coordinated by Dr. Manuela Deodato and Dr. Giacomo Rossettini.

Your contribution is extremely valuable and important. Thank you for taking the time to respond to this questionnaire for our profession.

If you have any questions, please contact:

Dr. Manuela Deodato (mdeodato@units.it)
Dr. Giacomo Rossettini (giacomo.rossettini@univr.it)

To provide your informed consent, click on the link for detailed explanations and to review the study information.
If you have any questions, don't hesitate to contact us.

LINK TO THE INFORMATION NOTE

By clicking on the link, I declare to have:

- Received detailed explanations regarding the study
- Reviewed the information note related to the study
- Had the opportunity to ask questions about the study

I am aware (to proceed, all answers must be selected):

- Of the risks and benefits associated with participating in this study
- That my participation is voluntary and I am free to withdraw at any time without explanation, and this will not affect my educational path or rights

Do you agree to participate in this study?

☐ Yes

☐ No

Are you a member of the Italian Physiotherapy Association (AIFI)?

☐ Yes

☐ No

PERSONAL DATA INFORMATION

To provide your consent for the processing of personal data, click on the link to review the information note. If you have any questions, do not hesitate to contact us.

LINK TO DATA PROCESSING INFORMATION

- [ ] I declare that I have reviewed the information regarding the processing of personal data
- [ ] I consent to the University of Trieste processing my personal data for the purposes and in the manner described

RESPONDENT CHARACTERISTICS

* 1. How old are you? [Complete numerically, e.g., 25]

* 2. What gender do you identify with? [Select]

- Male
- Female
- Non-binary
- I don’t want to answer

* 3. How many years have you been working as a physical therapist? [Select]

- < 5
- 6 - 10
- 11 - 20
- > 21

* 4. Which region of Italy do you work in? [Select]

- North
- Centre
- South

* 5. In which working sector do you practice? [Select]

- Public
- Private

* 6. What is your current professional status? [Select]

- Employed physiotherapist
- Free lancer

* 7. What is your workplace setting? [Select]

- Outpatients
- Hospitals
- Community

* 8. What kind of patients do you primarily rehabilitate? [Select]

- Pediatrics (< 18 years)
- Adults (18-65 years)
- Elderly (> 65 years)

* 9. What is your primary field of practice? [Select]

- Musculoskeletal
- Neurologic
- Oncologic-lymph
- Cardio-respiratory

○ Uro-gynaecological

* 10. How many hours do you work in a week? [Select]

○ 1-15 hours
○ 16-30 hours
○ 31-45 hours
○ 46-60 hours
○ more than 60 hours

* 11. What's your highest level of education [Select]

○ Bachelor's degree
○ Master's degree
○ Postgraduate diploma/certification
○ PhD

KNOWLEDGE AND USE

* 12. Have you ever heard about AI chatbots (e.g., ChatGPT, Microsoft Copilot, Google Gemini)?

- YES
- NO

* 13. If yes, in what context did you become aware of the existence of AI Chatbots? [Select]

- Social Media
- University lectures
- Friends/family/colleagues
- Traditional Media (TV/Newspapers)
- Scientific articles
- I have never heard of AI chatbots
- Other (specify)

* 14. Have you ever used AI chatbots in clinical practice? [Select]

- YES
- NO

* 15 If yes, how was your experience with AI Chatbots in clinical practice? [Select]

- Very negative
- Negative
- Neutral
- Positive
- Very positive
- I have never used an AI Chatbot for clinical purposes

* 16. If yes, for what purpose have you mainly used AI Chatbots in clinical practice? [You can select multiple answers]

- [ ] Medical history
- [ ] Clinical reasoning
- [ ] Functional Diagnosis
- [ ] Management of medical records
- [ ] Identify strategies for treating the patient's problems
- [ ] Identify empowerment strategies to encourage patient self-care
- [ ] Provide support in the interpretation of clinical data, test results or assessments
- [ ] Track down answers to use in educational interventions with the patient
- [ ] I have never used AI chatbots for clinical purposes
- [ ] Other (specify)

* 17. If yes, how often do you use AI chatbots for clinical purposes on average?

- ○ Never
- ○ Rarely (very low frequency)
- ○ Sometimes
- ○ Often (moderate frequency)
- ○ Always

* 18. How likely are you to use AI chatbots in the future for clinical purposes?

- ○ Never
- ○ Rarely (very low frequency)
- ○ Sometimes
- ○ Often (moderate frequency)
- ○ Always

* 19. How easy or difficult do you consider using AI Chatbots?

- ○ Very difficult
- ○ Difficult
- ○ Neither easy nor difficult
- ○ easy
- ○ Very easy

PERCEPTIONS

* 20. Do you think AI Chatbots are useful in the clinical practice?
[For each item, rate: Not at all useful, Not very useful, Somewhat useful, Very useful, Extremely useful]

	Not at all useful	Not very useful	Somewhat useful	Very useful	Extremely useful
Support during history taking	○	○	○	○	○
Support in clinical reasoning and functional diagnosis	○	○	○	○	○
Treatment planning for patients	○	○	○	○	○
Appointment schedule planning	○	○	○	○	○
Medical record management	○	○	○	○	○
Analyzing patient data and providing personalized therapeutic recommendations based on their medical history	○ ○	○ ○	○ ○	○ ○	○ ○
Processing invoices or bill payments	○	○	○	○	○
Identifying empowerment strategies to foster patient self-care	○	○	○	○	○
Tracking answers for use in educational interventions with the patient	○	○	○	○	○
Creating online content to give patients to perform exercises at home	○	○	○	○	○
Assist in content creation by generating ideas for social media posts and other marketing	○	○	○	○	○

* 21. What is your level of agreement with the following statements about AI Chatbots?

[For each item, rate: Strongly disagree, Disagree, neither agree nor disagree, Agree, Strongly agree]

	Strongly disagree	Disagree	Neither agree nor disagree	Agree	Strongly disagree
The use of AI Chatbots is helpful in providing access to accurate and up-to-date information	◯	◯	◯	◯	◯
AI chatbots can help me in my clinical practice	◯	◯	◯	◯	◯
AI chatbots are more useful than search engines (e.g., Google) or databases (e.g., PubMed)	◯	◯	◯	◯	◯

LIMITS AND BARRIERS

* 22. How much do you agree on the limits of AI Chatbots in clinical practice?
(For each item, rate: Strongly disagree, Disagree, neither agree nor disagree, Agree, Strongly agree)

	Strongly disagree	Disagree	Neither agree nor disagree	Agree	Strongly agree
Validity of inaccurate, out-of-date content	○	○	○	○	○
Privacy and ethical issues	○	○	○	○	○
Risk of dissemination of misinformation	○	○	○	○	○
Reduction in human interactions	○	○	○	○	○
Risk of self-diagnosis by patients	○	○	○	○	○
Reduction in quality of patient care	○	○	○	○	○
Harmful or incorrect clinical decisions	○	○	○	○	○
Lack of personalized treatments	○	○	○	○	○
