# Supplementary material for: Knowledge, use and perceptions of artificial intelligence Chatbots among Italian physiotherapists: an online cross-sectional survey
Source: Front Digit Health. 2025 Sep 8;7:1671521. doi: 10.3389/fdgth.2025.1671521 (PMC12450932; doi:10.3389/fdgth.2025.1671521)
Supplement: Supplementary file 2 [file Datasheet2.pdf]

INTRODUZIONE

Gentile Collega

Ti proponiamo di partecipare ad un progetto di ricerca rivolto ai fisioterapisti italiani.

Lo scopo di questo studio è quello di condurre un'indagine trasversale somministrando un questionario online per indagare la conoscenza, le barriere ed i limiti nell'utilizzo dei chatbot con intelligenza artificiale.
Esso verrà distribuito ai fisioterapisti italiani regolarmente iscritti all'Ordine professionale FNOFI appartenenti all'associazione tecnico scientifica rappresentativa della professione, ossia l'ASSOCIAZIONE ITALIANA FISIOTERAPIA (AIFI),
A tale proposito lo studio è stato illustrato al presidente AIFI dott. Simone Cecchetto per poterlo inoltrare alla mailing list degli iscritti.

L'obiettivo è di indagare la conoscenza, gli ambiti di applicazione, le potenzialità, i limiti e le questioni etiche riguardo l'utilizzo delle Artificial Intelligence Chatbots (es. ChatGPT, Microsoft Pilot e Google Gemini) nella pratica clinica del fisioterapista.
Le chiederemo di compilare un questionario che avrà una durata massima di 10 minuti.
La partecipazione allo studio è su base volontaria e non comporta alcun rischio specifico.
Tutti i dati raccolti saranno trattati e archiviati in maniera rigorosamente anonima ai sensi del F. Lgs 196/2003 e successive modifiche ed integrazioni in materia di trattamento dei dati personali.
Il suo contributo è estremamente prezioso ed importante. Grazie per il tempo che sta dedicando a rispondere al questionario per la nostra professione.

Per acquisire il suo consenso informato, cliccando sul link può ricevere spiegazioni esaustive e prendere visione della nota informativa relativa allo studio. Qualora avesse domande non esiti a contattarci.
LINK ALLA NOTA INFORMATIVA

* Per acquisire il suo consenso informato, cliccando sul link può ricevere spiegazioni esaustive e prendere visione della nota informativa relativa allo studio. Qualora avesse domande non esiti a contattarci.
Dichiaro di:

- ☐ Aver ricevuto spiegazioni esaustive in merito allo studio
- ☐ Aver preso visione della nota informativa relativa allo studio
- ☐ Aver avuto l'opportunità di fare domande in merito allo studio

* Sono consapevole (per proseguire è necessario selezionare tutte le risposte):

- [ ] Dei rischi e dei benefici che possono derivare dalla partecipazione a questo studio
- [ ] Che la mia partecipazione è volontaria, e di essere libero di potermi ritirare in qualunque momento senza dover dar spiegazioni e senza che il mio percorso formativo o i miei diritti ne siano condizionati

* Accetto di partecipare a questo studio?

- ◯ SI
- ◯ NO

* E' iscritto all'Associazione Italiana Fisioterapisti (AIFI)?

- ◯ Si
- ◯ No

INFORMATIVA TRATTAMENTO DATI PERSONALI

* Per acquisire il suo consenso al trattamento dei dati personali, cliccando sul link potrà prendere visione della nota informativa. Qualora avesse domande non esiti a contattarci.
LINK AL TRATTAMENTO DEI DATI

- ☐ DICHIARA di aver preso visione dell'Informativa per il trattamento dei dati personali
- ☐ PRESTA IL CONSENSO affinché l'Università degli Studi di Trieste tratti i propri dati personali per le finalità e secondo le modalità ivi descritte

CARATTERISTICHE DEL CAMPIONE

* 1. Quanti anni ha? [Completi numericamente es. 25]

* 2. In quale genere si identifica? [Selezioni]

- Maschio
- Femmina
- Non binario
- Altro/Preferisco non rispondere

* 3. Da quanti anni è fisioterapista? [Selezioni]

- <5
- 6-10
- 11-20
- >21

* 4. In che zona di Italia lavora? [Selezioni]

- Nord
- Centro
- Sud

* 5. In che settore lavora? [Selezioni]

- Pubblico
- Privato

* 6. Quale è il suo inquadramento professionale? [Selezioni]

- Dipendente
- Libero professionista

* 7. Quale è il suo ambito lavorativo? [Selezioni]

- Ambulatorio
- Ospedale
- Strutture assistenziali (casa di riposo, RSA)

* 8. Che tipologia di pazienti riabilita prevalentemente? [Selezioni]

- ◯ Pediatrici (< 18 anni)
- ◯ Adulti (18-65 anni)
- ◯ Anziani (> 65 anni)

* 9. Quale è il suo campo di intervento prevalente? [Selezioni]

- ◯ Muscoloscheletrico
- ◯ Neurologico
- ◯ Oncologico-linfologico
- ◯ Cardio-respiratorio
- ◯ Uro-ginecologico

* 10. Quante ore lavora a settimana? [Selezioni]

- ◯ 1-15
- ◯ 16-30
- ◯ 31-45
- ◯ 46-60
- ◯ >60

* 11. Quale è il suo livello di formazione più elevato? [Selezioni]

- ◯ Laurea Triennale
- ◯ Laurea Magistrale
- ◯ Master
- ◯ Dottorato di ricerca

CONOSCENZA E UTILIZZO

* 12. Ha mai sentito parlare di Artificial Intelligence Chatbots (es. ChatGPT, Microsoft Pilot e Google Gemini)? [Selezioni]

- SI
- NO

* 13. Se si, in che ambito è venuto a conoscenza dell'esistenza delle Artificial Intelligence Chatbots? [Selezioni]

- Social Media
- Lezioni Universitarie
- Amici/Famiglia/Compagni
- Media Tradizionali (Telegiornali/Giornali)
- Articoli scientifici
- Non ho mai sentito parlare di AI Chatbots

Altro (specificare)

* 14. Ha mai usato una Artificial Intelligence Chatbots nella pratica clinica? [Selezioni]

- SI
- NO

* 15. Se sì, come valuta la sua esperienza con le Artificial Intelligence Chatbots? [Selezioni]

- Molto negativa
- Negativa
- Neutra
- Positiva
- Molto Positiva
- Non ho mai utilizzo una Artificial Intelligence Chatbots per scopi clinici

* 16. Se sì, con quale funzione ha utilizzato maggiormente le Artificial Intelligence Chatbots? [E' possibile selezionare più risposte]

- [ ] Anamnesi
- [ ] Ragionamento clinico
- [ ] Diagnosi funzionale
- [ ] Gestione Cartelle cliniche
- [ ] Individuare strategie di trattamento dei problemi del paziente
- [ ] Individuare strategie di empowerment per favorire l'autocura del paziente
- [ ] Fornire supporto nell'interpretazione di dati clinici, risultati di test o valutazioni
- [ ] Rintracciare risposte da utilizzare negli interventi educativi con il paziente
- [ ] Non ho mai utilizzato le AI Chatbots per scopi clinici
- [ ] Altro (specificare)

* 17. Se si, quanto utilizza in media le Artificial Intelligence Chatbots per scopi clinici?

- ◯ Mai
- ◯ Raramente
- ◯ Qualche volta
- ◯ Spesso
- ◯ Sempre

* 18. Quanto è propenso/a ad utilizzare delle Artificial Intelligence Chatbots in futuro per scopi clinici?

- ◯ Mai
- ◯ Raramente
- ◯ Qualche volta
- ◯ Spesso
- ◯ Sempre

* 19. Quanto reputa sia il grado di difficoltà/facilità nell'utilizzo delle Artificial Intelligence Chatbots?

- ◯ Molto difficile
- ◯ Difficile
- ◯ Nè facile nè difficile
- ◯ Facile
- ◯ Molto facile

PERCEZIONI

* 20. Quanto crede siano utili le Artificial Intelligence Chatbots nella clinica?

	Per nulla utile	Non tanto utile	Abbastanza utile	Molto utile	Estremamente utile
Supporto durante l'anamnesi	◯	◯	◯	◯	◯
Supporto nel ragionamento clinico e diagnosi funzionale	◯	◯	◯	◯	◯
Pianificazione del trattamento dei pazienti	◯	◯	◯	◯	◯
Pianificazione dell'agenda degli appuntamenti	◯	◯	◯	◯	◯
Gestione delle cartelle cliniche	◯	◯	◯	◯	◯
Analizzare i dati dei pazienti e fornire raccomandazioni terapeutiche personalizzate in base alla loro storia medica	◯	◯	◯	◯	◯
Creazione di contenuti on-line da dare ai pazienti per eseguire gli esercizi a casa	◯	◯	◯	◯	◯
Assistere nella creazione di contenuti generando idee per post sui social media e altro materiale di marketing	◯	◯	◯	◯	◯
Elaborazione fatture o pagamenti di fatture	◯	◯	◯	◯	◯
Individuare strategie di empowerment per favorire l'autocura del paziente	◯	◯	◯	◯	◯
Rintracciare risposte da utilizzare negli interventi educativi con il paziente	◯	◯	◯	◯	◯

* 21. Quale è il suo grado di accordo con le seguenti affermazioni sulle Artificial Intelligence Chatbots?

	Molto disaccordo	Disaccordo	Nè in accordo nè in disaccordo	D'accordo	Molto d'accordo
L'utilizzo delle Artificial Intelligence Chatbots è utile per fornire accesso alle informazioni accurate e aggiornate	○	○	○	○	○
Le Artificial Intelligence Chatbots possono aiutarmi nella mia pratica clinica	○	○	○	○	○
Le Artificial Intelligence Chatbots sono più utili dei motori di ricerca (Es. Google) o delle banche dati (Es. Pubmed)	○	○	○	○	○

LIMITI E BARRIERE

* 22. Quanto si ritiene d’accordo sui limiti delle Artificial Intelligence Chatbots nella pratica clinica? [Selezioni]

	Molto disaccordo	Disaccordo	Nè in accordo nè in disaccordo	D'accordo	Molto d'accordo
Validità dei contenuti imprecisi, non accurati, non aggiornati	○	○	○	○	○
Problemi di Privacy ed Etici	○	○	○	○	○
Rischio di diffusione di informazioni errate	○	○	○	○	○
Riduzione delle interazioni umane	○	○	○	○	○
Rischio di autodiagnosi da parte dei pazienti	○	○	○	○	○
Riduzione della qualità dell’assistenza al pazienti	○	○	○	○	○
Decisioni cliniche dannose o sbagliate	○	○	○	○	○
Mancanza di trattamenti personalizzati	○	○	○	○	○
